# The new proposal classification criteria for juvenile spondyloarthropathies

**DOI:** 10.1186/1546-0096-12-S1-P45

**Published:** 2014-09-17

**Authors:** Metin Sezen, Kenan Barut, Cengizhan Açıkel, Ozgur Kasapcopur

**Affiliations:** 1 Pediatric Rheumatology, Istanbul University, Cerrahpasa Medical Faculty, Istanbul, Turkey; 2 Biostatistics, Gulhane Military Medical School , Ankara, Turkey

## Introduction

Juvenile spondyloarthropathies (JSpA) are a group of rheumatologic diseases with a disease onset before 16 years of age and are characterized with enthesitis, lower extremity oligoarthritis, involvement of the axial skeleton and HLA B27 positivity. The main problem in the classification of this group of diseases is the difficulty of diagnosis at disease onset and the difficulty to differentiate them from juvenile idiopathic arthritis.

## Objectives

The aim of our study was to evaluate the pre-determined classification criteria for children with JSpA and to develop new classification criteria.

## Methods

The study group consisted of 113 patients with the diagnosis of JSpA and 150 patients with juvenile idiopathic arthritis (JIA). All of the patients with JIA were diagnosed according to the ILAR criteria. Enthesitis related arthritis (ERA) and juvenile psoriatic arthritis (JPsA) were included into the JSpA group. Eligible criterion of atypical spondyloarthropathy criteria which can also be used for children, seronegative enthesopathy and arthropathy syndrome, enthesitis related arthritis, Garmisch- Partenkirchen, ESSG (European Spondyloarthropathies study Group), Amor, ASAS peripheral SpA, ASAS axial SpA criteria were applied to all of the enrolled patients. Odds ratios were determined for the variables of these criteria and for clinical findings in the classification of JSpA and JIA cases. Variables of the major findings of JSpA disease were determined by using probability analysis and clinical characteristics, Specificity, sensitivity and kappa values were determined for the new set diagnostic criteria proposed by us and for the pre-determined criteria in order to evaluate which diagnostic criteria were superior for the diagnosis of JSpA.

## Results

Major clinical variables of JSpA group of diseases by using pre-existing criteria are; oligoarthritis, enthesopathies, onset of disease after 6 or 10 years of age, inflammatory lumbar pain, hip arthritis, tarsometatarsal joint arthritis, male sex, rapid response to nonsteroidal anti-inflammatory drugs, sacroiliitis (MRI or radiography confirmed), HLA B27 positivity, limitation in lumber mobility test (below 4 cm), family history of SpA group of disease, dactylitis, psoriasis or presence of inflammatory bowel disease. All the variables had a kappa value over 0,8. The usefulness of all of the pre-determined criteria in children was low. The variables of the proposal new set criteria that were determined in our study were summarized below:**New set criteria: Major criterion**: Oligoarthritis, enthesopathy, disease onset after 6 years of age, inflammatory lumbar pain. **Minor criterion:** Hip arthritis, tarsometatarsal arthrtitis, male sex, NSAID response, sacroiliitis (in MR or radiography), HLA B27 positivity, limitation in schober test (<4cm), family history of SpA group of disease, dactylitis, psoriasis or presence of IBD. **Diagnosis:** 2 major + 3 minor or 3 major variables. The sensitivity, specificity and kappa value of this criteria were found as 90.3%, 90.7% and 0.807, respectively.

## Conclusion

We found that the pre-determined criteria were inadequate for the classification and diagnosis of juvenile spondyloarthropathies. We suggest that new set criteria proposed by us could be used in the diagnosis and classification of JSpA. However, neither the pre-determined criteria nor the new set criteria are adequate and efficacious for the classification and diagnosis of this disease.

## Disclosure of interest

None declared.

